# Blue-light fundus autofluorescence imaging of pigment epithelial detachments

**DOI:** 10.1038/s41433-022-02076-5

**Published:** 2022-05-17

**Authors:** Almut Bindewald-Wittich, Joanna Dolar-Szczasny, Sandrine H. Kuenzel, Leon von der Emde, Maximilian Pfau, Robert Rejdak, Steffen Schmitz-Valckenberg, Thomas Ach, Jens Dreyhaupt, Frank G. Holz

**Affiliations:** 1 Augenkompetenz Zentren, Heidenheim/Bopfingen, Germany; 2 Department of Ophthalmology, University of Bonn, Bonn, Germany; 3 Chair and Department of General and Pediatric Ophthalmology, Medical University of Lublin, Lublin, Poland; 4 John A. Moran Eye Center, Department of Ophthalmology & Visual Sciences, University of Utah, Salt Lake City, USA; 5 Institute of Epidemiology and Medical Biometry, Ulm University, Ulm, Germany

**Keywords:** Outcomes research, Retinal diseases, Macular degeneration

## Abstract

**Background:**

Pigment epithelial detachments (PEDs) occur in association with various chorioretinal diseases. With respect to the broad clinical spectrum of PEDs we describe fundus autofluorescence (FAF) characteristics of PEDs.

**Methods:**

Ninety-three eyes of 66 patients (mean age 71.9 ± 11.1) with uni- or bilateral PED ( ≥ 350 µm) were included in a retrospective cross-sectional study. PEDs were secondary to age-related macular degeneration (*n* = 79), central serous chorioretinopathy (*n* = 7), polypoidal choroidal vasculopathy (*n* = 2), pattern dystrophy (*n* = 3) or idiopathic PED (*n* = 2). FAF images were recorded using confocal scanning laser ophthalmoscopy (488 nm excitation wavelength, detection of emission >500 nm). Diagnosis of PED was confirmed using spectral-domain optical coherence tomography. A qualitative FAF grading system was established, and grading was performed by two independent readers.

**Results:**

PEDs showed highly variable characteristics on FAF imaging. FAF within the area of PED was found to be irregular/granular (*n* = 59, 63.4%), increased (*n* = 28, 30.1%), decreased (*n* = 3, 3.2 %), or normal (*n* = 3, 3.2%). Accompanying FAF changes included condensation of macular pigment (*n* = 67, 72.0%), focally increased FAF at the PED apex (*n* = 14, 15.1%) or elsewhere (*n* = 52, 55.9%), focally decreased FAF (*n* = 23, 24.7%), a cartwheel-like pattern (*n* = 10, 10.8%), a doughnut sign (*n* = 6, 6.5%), and a halo of decreased FAF encircling the PED (completely *n* = 20, 21.5% or incompletely *n* = 20, 21.5%).

**Conclusions:**

PEDs show a variety of abnormal patterns on FAF imaging. These changes in FAF signals may be secondary to morphological and metabolic alterations within corresponding retinal layers and do not necessarily correspond with the underlying PED subtype or a specific pathology.

## Introduction

Retinal pigment epithelial detachments (PEDs) are characterized by a separation of the retinal pigment epithelium (RPE) monolayer with its basal lamina from the inner collagenous zone of Bruch’s membrane (BM) resulting in a flat to dome-shaped elevation of the RPE with typically well-demarcated margins. PEDs are a non-specific clinical sign and may occur in association with various chorioretinal diseases with the most common aetiology being age-related macular degeneration (AMD). Other causes of PED include central serous chorioretinopathy (CSCR), polypoidal choroidal vasculopathy (PCV), pachychoroid syndrome, retinal dystrophies, systemic diseases, and idiopathic PED. Various pathophysiological concepts have been brought forward regarding the pathogenesis of PEDs [1–4]. Age-related changes of BM and within the sub-RPE space are discussed in the context of AMD. Thereby, ageing of BM with thickening of the inner collagenous layer, collagen cross-linking and accumulation of debris including hydrophobic lipids may result in decreased hydraulic conductivity and, consequently, lead to reduced transepithelial fluid flow [5–9]. The amount of trapped fluid in the sub-RPE space may be further increased by a passive inflow of water due to a modified osmolarity based on changes in extracellular matrix proteins [10, 11]. Furthermore, reduced oxygen transport may lead to hypoxia with subsequently increased secretion of vascular endothelial growth factor and risk of formation of hyperpermeable choroidal neovascularization underneath the RPE, which then may directly cause PED via exudation. Type 1 macular neovascularization may by itself induce a PED due to hyperpermeability and accumulation of sub-RPE fluid. In contrast, PEDs in CSCR possibly derive from choroidal dysfunction and increased permeability of choroidal vessels in association with exogenous and/or endogenous factors including an overactivation of mineralocorticoid receptors [12].

Since the seminal review of Gass in 1967 [13] conventional and recent imaging modalities have contributed to a better characterization of PEDs [14]. Previous clinical classifications of PEDs were based on funduscopic, and fluorescence angiographic characteristics [15–19]. Recent studies include findings in spectral domain optical coherence tomography (SD-OCT) and OCT angiography [20–24]. Moreover, multimodal imaging modalities are complemented by further advances of digital imaging devices and allow for assessment of PEDs using fundus autofluorescence (FAF) imaging.

FAF imaging represents a non-invasive tool to evaluate endogenous fluorophores of the ocular fundus [25–30]. FAF mainly derives from lipofuscin (LF) and melanolipofuscin (MLF) at the level of the RPE as shown by spectrometric investigations and high-resolution microscopy [31–33]. Other possible fluorophores within the outer retina and in the subretinal space, as well as absorbing factors need to be considered when interpreting FAF images.

Refined phenotypic FAF classifications have been established for intermediate and atrophic late AMD [27, 34–36]. FAF imaging in PEDs due to AMD was initially described by von Rückmann et al. in 21 eyes [37]. They reported that PEDs older than 6 months reveal a mild, diffuse, increased FAF signal corresponding with the detached area, but no further sub-classification of PEDs was brought forward. Karadimaz and Bouzas investigated FAF changes in serous and drusenoid PEDs in 14 AMD eyes and reported an increased FAF signal in the area of serous vascularized and avascular PEDs, whereas in drusenoid PEDs, FAF was either increased or decreased [38]. A systematic description of variations in FAF in association with PEDs of different origin has been lacking so far. Prior observations obtained during the prospective multicentre natural history FAM study (***F***undus ***A***utofluroescence in Age-related ***M***acular Degeneration) suggested that FAF imaging in presence of PED gives additional information over and above conventional fundus photography or fluorescence angiography and that there is no uniform FAF phenotype of PEDs [39]. Here, we sought to describe phenotypic FAF changes in the area of PEDs of different causes.

## Materials and methods

Patients with uni- or bilateral PED were included in this retrospective cross-sectional study. PED was defined as a separation of the RPE from the BM. Diagnosis of PED was confirmed by SD-OCT. Eyes with detection of a PED at a single point in time and a documented ophthalmologic examination, that encompassed good-quality FAF imaging, fluorescein angiography, and SD-OCT imaging, were included. Availability of indocyanine green angiography was facultative. Exclusion criteria were insufficient image quality for any reason, and a lack of adequate and complete image data sets. Eyes with complete masking of the PED due to complex additional findings were excluded. Even if there is no established uniform definition of the horizontal dimension of a PED with prior studies tending to larger sizes [2, 19, 40–43], we based our data on the definition applied in the age-related eye disease study (AREDS). In AREDS Report No. 28 the narrowest diameter of drusenoid PEDs is determined not to be less than 350 µm [42]. This minimum dimension was taken as a basis for all subtypes of PED in our study as smaller lesions are unlikely to result in definite FAF changes. In presence of multiple PEDs in one eye analysis focused on the largest PED. The following patient characteristics were documented: sex (male/female), age, affected eye, and best corrected visual acuity (logMAR). The research methods and analysis were in accordance with the Tenets of the World Medical Association Declaration of Helsinki. Patients were only seen for their routine examinations at the Department of Ophthalmology of the University Hospital of Bonn, Germany, between January 2010 and June 2020, and data was analysed after irreversible anonymization.

Digital FAF images were acquired following internal patient care standards of the Department of Ophthalmology of the University Hospital of Bonn, Germany [36]. After pupil dilation with tropicamide 1.0% and phenylephrine 2.5%, short wavelength FAF images (excitation wavelength 488 nm, detection of the emitted light >500 nm using a barrier filter) were obtained using a confocal scanning laser ophthalmoscope (SPECTRALIS HRA + OCT2, Heidelberg Engineering, Heidelberg, Germany). In most cases, at least 15 averaged frames were imaged. If appropriate, the automatic real time tracking was used. Images with 30- or 55-degrees field of view encompassed the macula. SD-OCT volume scans and infrared reflectance images were acquired using the same imaging device. The SD-OCT scan pattern (e.g., area covered, number of B-scans) varied between the patients because of the retrospective nature of this study. Fluorescein and indocyanine green angiography were performed with 30- or 55-degrees field of view.

FAF images were qualitatively analysed for changes differing from the normal background autofluorescence signal. SD-OCT images were graded for distinct parameters. A grading system was established including the diagnosis, PED type, localization of the PED, FAF changes within the area of PED, concomitant findings, and OCT criteria like the PED content and morphological changes overlying the detached area [Tables 1–3]. Images were assessed by two independent graders that were masked regarding clinical examination, clinical diagnosis, and therapy. In case of discrepancy the final decision was made by an experienced senior grader.

**Table 1:** Frequencies of PED subtypes for all study eyes and in association with the main diagnoses.

PED subtypes	Total (*n* = 93)		AMD (*n* = 79)		CSCR (*n* = 7)		Pattern dystrophy (*n* = 3)		PCV (*n* = 2)		Idiopathic PED (*n* = 2)	
	*n*	%*	*n*	% *	*n*	%*	*n*	%*	*n*	%*	*n*	%*
Drusenoid PED	41	44.1	39	41.9	–	–	2	2.2	–	–	–	–
Serous PED	18	19.4	10	10.8	5	5.4	–	–	1	1.1	2	2.2
Vascularized PED	12	12.9	10	10.8	1	1.1	–	–	1	1.1	–	–
Mixed PED	20	21.5	18	19.4	1	1.1	1	1.1	–	–	–	–
Haemorrhagic PED	2	2.2	2	2.2	–	–	–	–	–	–	–	–

Pigment epithelial detachment (PED), age-related macular degeneration (AMD), polypoidal choroidal vasculopathy (PCV), central serous chorioretinopathy (CSCR).

^*^Percent of all study eyes.

**Table 2:** Frequencies of FAF changes (A) and concomitant findings (B) associated with PEDs.

A. Frequencies of FAF changes and their correlation with PED subtypes (in all study eyes)														
FAF changes within the area of PED	Total (*n* = 93)		AMD eyes (*n* = 79)		Drusenoid PED (*n* = 41)		Serous PED (*n* = 18)		Vascularized PED (*n* = 12)		Mixed PED (n = 20)		Haemorrhagic PED (*n* = 2)	
	*n*	%*	n	%**	*n*	%*	n	%*	*n*	%*	*n*	%*	*n*	%*
Irregular/granular FAF signal	59	63.4	50	63.3	24	25.8	10	10.8	8	8.6	16	17.2	1	1.1
Homogeneously increased FAF	28	30.1	25	31.7	16	17.2	5	5.4	3	3.2	3	3.2	1	1.1
Homogeneously decreased FAF	3	3.2	3	3.8	–	–	1	1.1	1	1.1	1	1.1	*–*	–
Normal FAF	3	3.2	1	1.3	1	1.1	2	2.2	–	–	–	–	*–*	–
Condensation of macular pigment	67	72.0	61	77.2	38	40.9	7	7.5	6	6.5	15	16.1	1	1.1
Focally increased FAF elsewhere	52	55.9	46	58.2	25	26.9	8	8.6	7	7.5	12	12.9	*–*	*–*
Focally increased FAF at apex of PED	14	15.1	11	13.9	5	5.4	6	6.5	1	1.1	2	2.2	*–*	*–*
Focally decreased FAF (RPE atrophy)	23	24.7	20	25.3	6	6.5	6	6.5	4	4.3	7	7.5	*–*	*–*
Cartwheel-like FAF pattern	10	10.8	7	8.9	7	7.5	1	1.1	–	–	2	2.2	*–*	*–*
Doughnut sign	6	6.5	5	6.3	3	3.2	2	2.2	1	1.1	–	–	*–*	*–*
Halo of decreased FAF, complete	20	21.5	17	21.5	10	10.8	5	5.4	–	–	5	5.4	*–*	*–*
Halo of decreased FAF, incomplete	20	21.5	20	25.3	13	14.0	3	3.2	–	–	4	4.3	*–*	*–*
**B. Frequencies of concomitant findings and their association with PED subtypes (in AMD eyes)**														
**Concomitant findings**	**Total (*n*** = **93)**		**AMD eyes (*n*** = **79)**		**Drusenoid PED (*n*** = **39)**		**Serous PED (*n*** = **10)**		**Vascularized PED (*n*** = **10)**		**Mixed PED (*n*** = **18)**		**Haemorrhagic PED (*n*** = **2)**	
	***n***	%*	***n***	%**	***n***	%**	***n***	%**	***n***	%**	***n***	%**	***n***	%**
Sub-RPE haemorrhage	2	2.2	2	2.5	–	–	–	–	–	–	–	–	2	2.5
Subretinal haemorrhage	8	8.6	8	10.1	–	–	–	–	2	2.5	5	6.3	1	1.3
Intraretinal haemorrhage	3	3.2	3	3.8	–	–	–	–	–	–	3	3.8	–	–
Geographic atrophy	11	11.8	10	12.7	3	3.8	6	7.6	–	–	1	1.3	–	–
RPE tear	3	3.2	3	3.8	–	–	2	2.5	–	–	–	–	1	1.3
Soft drusen	58	62.4	56	71.0	39	49.4	6	7.6	6	7.6	5	6.3	-	–
Subretinal drusenoid deposits	15	16.1	15	19.0	1	12.7	2	2.5	4	5.1	7	8.9	1	1.3
Cuticular Drusen	44	47.3	42	53.2	26	32.9	4	5.1	6	7.6	6	7.6	–	–
Hard drusen	5	5.4	4	5.1	2	2.5	–	–	–	–	1	1.3	1	1.3

Fundus autofluorescence (FAF), pigment epithelial detachment (PED), retinal pigment epithelium (RPE).

^*^Percent of all study eyes.

^**^Percent of AMD eyes.

**Table 3:** Correlation of SD-OCT criteria with the PED subtype and the FAF pattern in the area of PED in all study eyes.

A. SD-OCT criteria versus PED subtype										
	Drusenoid (*n* = 41)		Serous (*n* = 18)		Vascularized (*n* = 12)		Mixed (*n* = 20)		Haemorrhagic (*n* = 2)	
	*n*	%*	*n*	%*	*n*	%*	*n*	%*	*n*	%*
**Content of PED in SD-OCT**										
Predominantly optically empty (*n* = 27)	4	4.3	15	16.1	–	–	8	8.6	–	–
Predominantly hyperreflective (*n* = 44)	35	37.6	3	3.2	3	3.2	2	2.2	1	1.1
Hyperreflective speckled (*n* = 10)	2	2.2	–	–	1	1.1	6	6.5	1	1.1
Onion sign (*n* = 12)	–	–	–	–	8	8.6	4	4.3	–	–
**Hyperreflective material**										
Intraretinal sloughed RPE (*n* = 62)	31	33.3	10	10.8	7	7.5	13	14.0	1	1.1
Intraretinal hyperreflective foci (*n* = 66)	31	33.3	9	9.7	8	8.6	16	17.2	2	2.2
Intraretinal vitelliform material (*n* = 29)	14	15.1	8	8.6	3	3.2	4	4.3	–	–
Basolateral RPE shedding (*n* = 47)	18	19.4	5	5.4	9	9.7	13	14.0	2	2.2
**PED contour**										
Predominantly smooth (*n* = 74)	39	42.0	18	19.4	3	3.2	13	14.0	1	*1.1*
Predominantly wrinkled (*n* = 19)	2	2.2	–	–	9	9.7	7	7.5	1	*1.1*
**Fluid**										
SRF covering the PED partially (*n* = 34)	8	8.6	5	5.4	7	7.5	12	12.9	2	*2.2*
SRF covering the PED completely (*n* = 6)	–	–	2	2.2	1	1.1	3	3.2	–	*–*
Intraretinal fluid (*n* = 14)	2	2.2	1	1.1	2	2.2	9	9.7	–	–
**B**. **SD-OCT criteria versus FAF signal in the area of PED**										
	**Irregular/ granular (*n*** = **59)**		**Homogeneously increased (*n*** = **28)**		**Homogeneously decreased (*n*** = **3)**		**Normal (*n*** = **3)**			
	***n***	%*	***n***	%*	***n***	%*	***n***	%*		
**Content of PED in SD-OCT**										
Predominantly optically empty (*n* = 27)	16	17.2	7	7.5	2	2.2	2	2.2		
Predominantly hyperreflective (*n* = 44)	26	28.0	17	18.3	–	–	1	1.1		
Hyperreflective speckled (*n* = 10)	7	7.5	2	2.2	1	1.1	–	–		
Onion sign (*n* = 12)	10	10.8	2	2.2	–	–	–	–		
**Hyperreflective material**										
Intraretinal sloughed RPE (*n* = 62)	42	45.2	13	14.0	2	2.2	–	–		
Intraretinal hyperreflective foci (*n* = 66)	42	45.2	14	15.1	2	2.2	1	1.1		
Intraretinal vitelliform material (*n* = 29)	20	21.5	7	7.5	–	–	–	–		
Basolateral RPE shedding (*n* = 47)	36	38.7	7	7.5	3	3.2	1	1.1		
**PED contour**										
Predominantly smooth (*n* = 74)	43	46.2	25	26.9	3	3.2	3	3.2		
Predominantly wrinkled (*n* = 19)	16	17.2	3	3.2	–	–	–	–		
**Fluid**										
SRF covering the PED partially (*n* = 34)	25	26.9	8	8.6	1	1.1	–	*–*		
SRF covering the PED completely (*n* = 6)	4	4.3	2	2.2	–	–	–	*–*		
Intraretinal fluid (*n* = 14)	12	12.9	1	1.1	1	1.1	–	*–*		

Pigment epithelial detachment (PED), spectral domain optical coherence tomography (SD-OCT), fundus autofluorescence (FAF), subretinal fluid (SRF).

^*^Percent of all study eyes.

Statistical analysis was performed using SAS, version 9.4 under Windows (SAS Institute, Chicago, IL, USA). Continuous variables were summarized as mean and standard deviation (SD) or median and interquartile range as appropriate, and the range was calculated. Categorical variables were presented as absolute and relative frequencies. Interobserver variability was calculated using the kappa coefficient (*κ*). Furthermore, the proportion of agreement including 95% confidence interval (CI) between the two graders was evaluated. Regarding the explorative nature of this study, results from the statistical tests should not be interpreted as confirmatory but rather as exploratory. Adjustment for multiple testing was not performed.

## Results

A total of 143 eyes of 87 patients with the clinical diagnosis of PED were analysed. Ninety-three eyes of 66 patients (mean age 71.9 ± 11.1 years, range 41.3–93.8 years) with uni- or bilateral PED met the inclusion criteria for this retrospective study. Thirty-seven (56.1%) were female. Median best corrected visual acuity was 0.3 logMAR (range 0.0–2.3 logMAR; interquartile range 0.1–0.4). Besides FAF imaging, SD-OCT, and fluorescein angiography, 24 patients (36.4%) had undergone additional indocyanine green angiography. Study eyes included 49 right eyes (52.7%) and 44 left eyes (47.3%). In 84 eyes (90.3 %) the PED involved the fovea (interobserver variability: proportion of agreement: 90 out of 93 = 96.8%, 95% CI [90.9%–99.3%]; *κ* = 0.82, CI [0.63–1.00]). Seventy eyes (75.3%) presented with monofocal PED, whereas multifocal PEDs were found in 23 eyes (24.7%) (interobserver variability: proportion of agreement: 68 out of 93 = 73.1% [62.9%–81.8%]; *κ* = 0.37 [0.19–0.55]).

In 79 eyes (85.0%) of 55 patients (83.3%) PED occurred in association with AMD, whereby 50 of 79 eyes (63.3%) had non-exudative/non-neovascular AMD, 5 of 79 eyes (6.3%) non-exudative neovascular AMD, and 24 of 79 eyes (30.4%) exudative neovascular AMD. Suspicion of a quiescent macular neovascularization without any signs of exudation was classified as non-exudative neovascular AMD, although reliable confirmation by OCT angiography was not available [44–46]. Other diagnoses included CSCR (7 eyes [7.5%] of 6 patients [9.1%]), pattern dystrophy (3 eyes [3.2%] of 2 patients [3.0%]), PCV (2 eyes [2.2%] of 2 patients [3.0%]), and idiopathic PED (2 eyes [2.2%] of 1 patient [1.5%]). The agreement between the two readers evaluating the main diagnoses was 89 out of 93 = 95.7% [98.4%–98.8%]; *κ* = 0.85 [0.71–0.98]. Fifty of 93 eyes (53.8%) were treatment-naïve at the date of examination. In 4 eyes the therapy status was unknown. 15 of 24 eyes with exudative neovascular AMD had received previous anti-VEGF therapy. Previous anti-VEGF therapy was documented in 1 eye with actual non-exudative neovascular AMD, and in 11 eyes with actual as non-exudative/non-neovascular classified AMD, but thereof only 4 eyes with a previous anti-VEGF therapy during the last 12 months. Three eyes with CSCR, 3 eyes with pattern dystrophy, and 2 eyes with PVC had also a previous anti-VEGF therapy. Two patients had a positive medical history regarding oral eplenerone therapy.

Among all eyes, secondary findings that did not significantly interfere with FAF changes due to PED included epiretinal membranes in 9 eyes (9.7%), papilledema in 1 eye (1.1%), peripapillary pachychoroid syndrome in 1 eye (1.1%), and a choroidal nevus in 1 eye (1.1%) (interobserver variability: proportion of agreement: 91 out of 93 = 98.0% [92.5%–99.7%]; *κ* = 0.91 [0.80–1.00]).

PED subtypes were classified into drusenoid, mixed, serous, vascularized, and haemorrhagic PEDs (Table 1) (proportion of agreement: 72 out of 93 = 77.4% [67.6%–85.4%]; *κ* = 0.67 [0.55–0.79]). Drusenoid PEDs were the most frequent PED type (41 out of 93 eyes [44.1%]), reflecting the high proportion of eyes with non-exudative/non-neovascular AMD within all included study eyes. They were associated with AMD in 39 eyes and with macular dystrophy in 2 eyes. Serous PEDs (18 out of 93 eyes [19.4%]) were associated with PCV (*n* = 1), CSCR (*n* = 5), idiopathic PED (*n* = 2), non-exudative/non-neovascular AMD (*n* = 8), and neovascular AMD (MNV type 1, *n* = 2). Vascularized PEDs (12 out of 93 eyes [12.9%]) were associated with AMD (*n* = 10), PCV (*n* = 1), and CSCR (*n* = 1). Mixed PEDs (20 out of 93 eyes [21.5%]) occurred in eyes with AMD (*n* = 18), CSCR (*n* = 1), and macular dystrophy (*n* = 1).

**Fig. 1:**
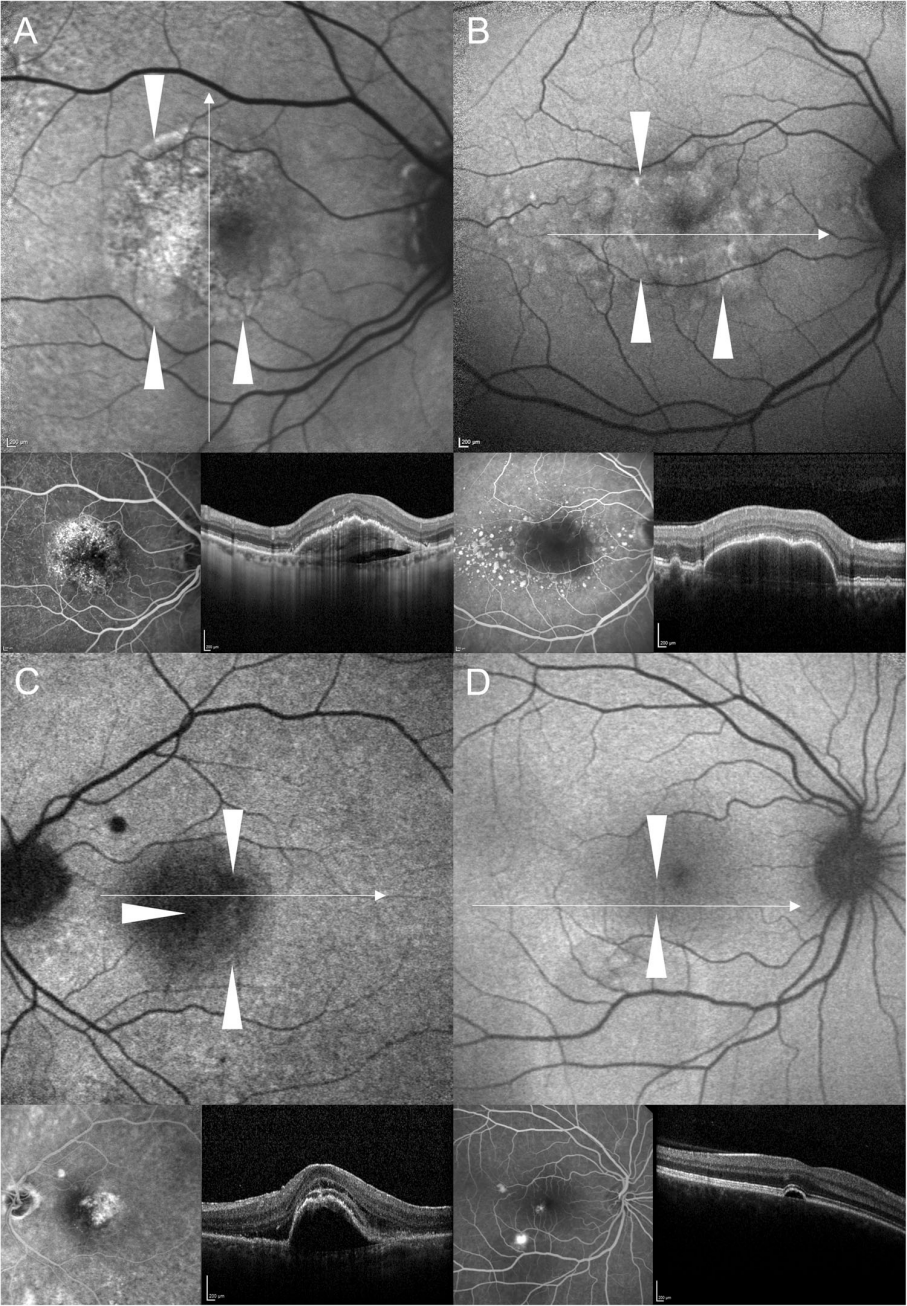
Fundus autofluorescence (FAF) images showing the four main types of FAF changes within the area of pigment epithelial detachment (PED): A Irregular/granular FAF changes. B Increased FAF. C Decreased FAF. D Normal FAF. **A** FAF image of a 78-years-old female with mixed-type PED due to neovascular age-related macular degeneration (AMD) showing irregular/granular FAF changes with both, increased FAF and spots of decreased FAF. SD-OCT shows an optically empty cleft under the vascularized part of the PED, sloughed RPE, and intraretinal hyperreflective foci. **B** Drusenoid PED with increased FAF within the area of PED in a 56-years-old patient with AMD. Apart from a large, subfoveal PED, there are soft confluent drusen and smaller PEDs, which are also characterized by increased FAF. There is no evidence of macular neovascularisation in fluorescein angiography. **C** This FAF image shows decreased FAF in the area of a PED (76-years-old male patient with mixed macular neovascularisation due to AMD). SD-OCT reveals SRF at the margin of the PED as well as overlying the PED. **D** FAF image of a 41-years-old male patient with central serous chorioretinopathy. The small juxtafoveal serous PED does not show essential FAF changes, whereas in the lower part of the FAF image increased FAF demarcates an area with SRF resulting from an expansile dot in FA. In SD-OCT overlying retinal layers are intact. White wedges mark the PED dimension in FAF images. White arrows in FAF images mark the position of the SD-OCT scan. Corresponding FA and SD-OCT scans are shown below the FAF images.

The following FAF changes were identified in association with PEDs (Table 2A):

In descending frequency, FAF within the area of PED was irregular/granular (with fractions of decreased, increased, and/or normal FAF), homogeneously increased, homogeneously decreased, or normal. The majority of PEDs in our study showed an irregular/granular FAF signal (*n* = 59, 63.4%) followed by homogeneously increased FAF (*n* = 28, 30.1%), homogeneously decreased FAF (*n* = 3, 3.2%), and normal FAF (*n* = 3, 3.2%) (Fig. 1A–D) (interobserver variability: proportion of agreement: 84 out of 93 = 90.3% [82.4%–95.5%]; *κ* = 0.82 [0.71–0.93]).

**Fig. 2:**
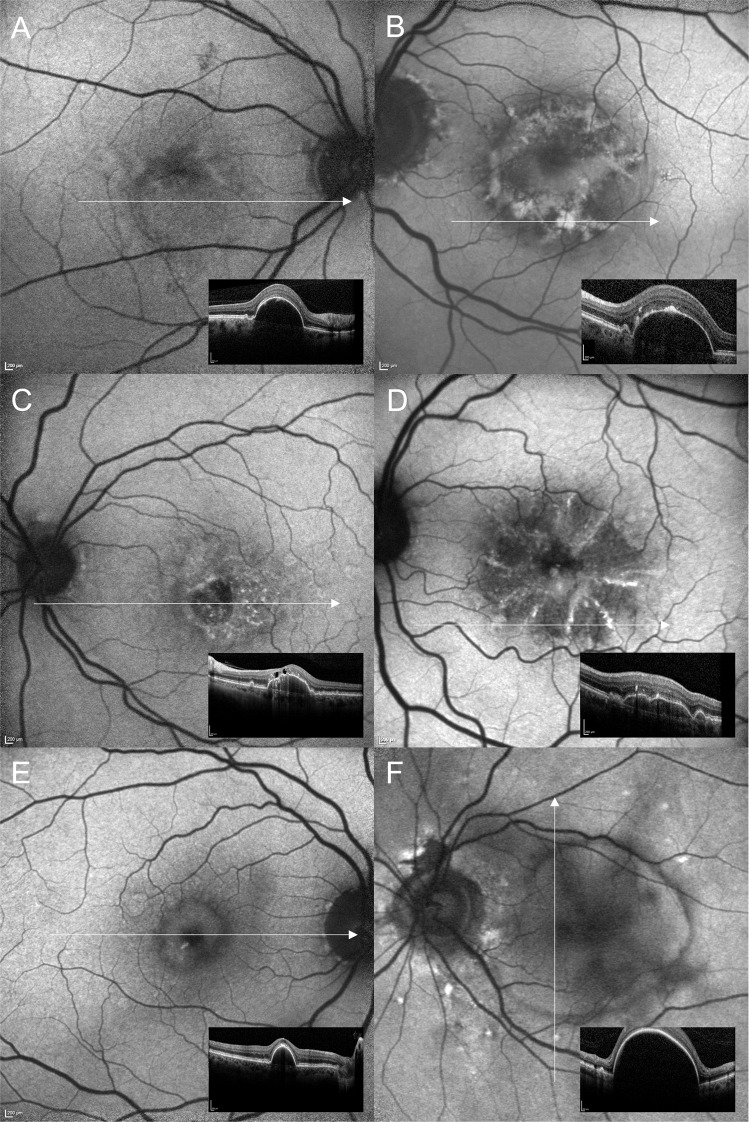
Facultatively accompanying fundus autofluorescence (FAF) changes within the area of pigment epithelial detachment (PED): A Condensation/displacement of macular pigment. B Focally increased FAF. C Focally decreased FAF due to RPE atrophy. D Cartwheel-like FAF pattern. E Doughnut sign. F Halo of decreased FAF. **A** FAF image of a mixed PED (serous-vascularized) of a 57.9-years-old female with macular neovascularization type 1 due to age-related macular degeneration (AMD). The inferior part of the PED presents with increased FAF, whereas FAF is decreased within the superior portion of the PED (in presence of subretinal fluid, not shown). The normal macular pigment distribution seems to be disturbed especially in presence of increased FAF. **B** Focally increased FAF may occur within the area of PED, at the apex, and elsewhere. In this left eye of a 68.9-years-old male with a large serous PED due to non-neovascular AMD focally increased FAF is associated with anterior RPE migration and an acquired vitelliform lesion in OCT. **C** Amongst the irregular/granular FAF pattern in this eye of a 63.9-years-old male patient there is an area with decreased FAF due to RPE atrophy in presence of a drusenoid PED. Fluorescein angiography (not shown) did not reveal any neovascularization. Besides intraretinal degenerative cysts, SD-OCT shows sloughed RPE, intraretinal hyperreflective foci, and basolateral RPE shedding. **D** FAF image of a 78.2-years-old female with drusenoid PED showing a cartwheel-like increased FAF pattern with centrifugal lines of increased FAF. **E** In case of this drusenoid PED (75.5-years-old female with intermediate AMD) FAF is focally decreased at the PED apex (“doughnut sign”) despite OCT shows anterior RPE migration. **F** The margin of this large serous PED is delineated by decreased FAF (74.9-years-old female patient with polypoidal choroidal vasculopathy). There is some FAF blurring according to anterior opacities.

Furthermore, facultatively accompanying FAF changes within the area of PED were frequently described as a condensation or a displacement of macular pigment and a focally increased FAF signal (at the PED apex or elsewhere overlying the PED). In AMD eyes 42 of 79 eyes (84%) with non-neovascular/non-exudative AMD and 17 of 24 (70.8%) eyes with exudative neovascular AMD showed a condensation of macular pigment. About a quarter of all study eyes showed focally decreased FAF due to focal RPE atrophy. Only few eyes showed a broad ring of moderate increased FAF with decreased FAF at the centre of PED (doughnut sign) or a cartwheel-like FAF pattern (interobserver variability: proportion of agreement: 510 out of 558 = 91.4% [88.8%–93.6%]). The cartwheel-like pattern was found in 3 eyes with pattern dystrophy and 7 eyes with non-neovascular/non-exudative AMD and did not occur in eyes with neovascular AMD. Some PEDs were surrounded by a margin of decreased FAF, which can encircle the PED completely or incompletely (Fig. 2) (interobserver variability: proportion of agreement: 78 out of 93 = 83.9% [74.8%–90.7%]; *κ* = 0.72, [0.60–0.85]). Associations of these FAF changes with the graded PED subtypes are summarized in Table 2A. The sub-analysis did not reveal a significant association between the FAF pattern and a specific PED type. Similarly, sub-analysis of the AMD subtypes (non-neovascular/non-exudative, non-exudative/neovascular, exudative neovascular) showed comparable results with the irregular/granular FAF pattern being the most frequent for all AMD subtypes.

Evaluation of the PED content appearance in SD-OCT scans resulted in 4 different types: predominantly optically empty (*n* = 27, 29.0%), predominantly hyperreflective (*n* = 44, 47.3%), hyperreflective speckled (*n* = 10, 10.8%), and with organized hyperreflective bands (“onion sign” [14, 47], *n* = 12, 12.9%, interobserver variability: proportion of agreement 77 out of 93 = 82.8% [73.6%; 89.8%]; *κ* = 0.74 [0.62–0.85]).

Serous, avascular PEDs tended to appear optically empty (15 out of 18 eyes [83.3%]), whereas drusenoid PEDs were mainly associated with internal hyperreflective OCT signals (35 out of 41 eyes [85.4%]). There was no predominant PED content associated with mixed PEDs. The onion sign indicated a vascularized portion of the PED (8 out of 12 eyes with vascularized PED, 66.7%; 4 out of 20 eyes with mixed PED, 20%) and was not found in eyes with drusenoid or serous PED (Table 3A). The association of the PED content in SD-OCT with the predominant FAF pattern in the area of PED is given in Table 3B. A homogeneously increased (17 out of 28 eyes [60.7%]) and an irregular/granular FAF signal (26 out of 59 eyes [44.1%]) was preferably associated with a predominantly hyperreflective signal within the PED in SD-OCT scans. The onion sign in SD-OCT mainly occurred in presence of an irregular/granular FAF pattern (10 out of 12 eyes [83.3%]).

SD-OCT scans were evaluated regarding hyperreflective material overlying the PED and revealed a great proportion of eyes with intraretinal hyperreflective material reflecting associated intraretinal morphological changes in presence of PED. In 62 eyes (66.7%) intraretinal sloughed RPE was found as a sign of anterior migration (interobserver variability: proportion of agreement: 82 out of 93 = 88.2% [79.8%–94.0%]; *κ* = 0.75 [0.62–0.89]) [48]. Sixty-six eyes (71.0%) featured intraretinal hyperreflective foci (interobserver variability: proportion of agreement: 83 out of 93 = 0.893% [81.1%–94.7%]; *κ* = 0.76 [0.62–0.90]), and circumscribed vitelliform material was present in 29 eyes (31.2%) (interobserver variability: proportion of agreement: 79 out of 93 = 85.0% [76.0%–91.5%]; *κ* = 0.66 [0.0.50–0.82]). Hyperreflective material was especially associated with the irregular/granular FAF pattern. 67.7 % of eyes with intraretinal sloughed RPE, 67.7% of eyes with intraretinal hyperreflective foci, 68.0% of eyes with intraretinal vitelliform material and 76.6% of eyes with basolateral RPE shedding were graded with irregular/granular FAF. By the majority, the PED contour turned out to be predominantly smooth in 74 eyes (79.6%), whereas in 19 eyes (20.4%) the PED contour was predominantly wrinkled (interobserver variability: proportion of agreement: 79 out of 93 = 90.3% [82.4%–95.5%]; *κ* = 0.70 [0.0.52–0.88]). Basolateral RPE shedding occurred in 47 eyes (50.5%) (interobserver variability: proportion of agreement: 76 out of 93 = 81.7% [72.4%–89.0%]; *κ* = 0.64 [0.49–0.79]). PEDs were completely covered by subretinal fluid (SRF) in 6 eyes (6.5%) and partially covered by SRF in 34 eyes (36.6%) (interobserver variability: proportion of agreement: 171 out of 186 = 91.9% [87.1%–95.4%]; *κ* = 0.76 [0.64–0.88]). Fourteen eyes (15.1%) showed intraretinal fluid overlying the PED (interobserver variability: proportion of agreement: 87 out of 93 = 93.6% [86.5%–97.6%]; *κ* = 0.73 [0.0.53–0.93]). The association of these SD-OCT criteria with PED subtypes and main FAF changes is given in Table 3A, B.

General concomitant findings that may change the FAF signal were identified as sub-RPE/subretinal/intraretinal haemorrhage, geographic atrophy, RPE tear, and different drusen types (soft drusen, subretinal drusenoid deposits, cuticular drusen, and hard drusen). These concomitant findings did not occur in eyes with PCV, CSCR and idiopathic PED, and only sporadic in eyes with pattern dystrophy. In eyes with AMD (*n* = 79) they were registered as follows (Table 2B) (interobserver variability: proportion of agreement 661 out of 711 = 93.0% [90.8% - 94.7%]):

- Haemorrhages: Sub-RPE haemorrhage (*n* = 2, 2.5%), subretinal haemorrhage (*n* = 8, 10.1%), intraretinal haemorrhage (*n* = 3, 3.8%). All kinds of haemorrhages occurred in neovascular AMD only. Haemorrhage did not occur in presence of drusenoid or serous PED subtypes, but in presence of a vascularized, mixed, or haemorrhagic PED.
- Geographic atrophy (*n* = 10, 12.7%) was mainly associated with drusenoid and serous PEDs and found in eyes with partially resolved PED and therefore subsequent transition to geographic atrophy.
- RPE tear: Only 3.8% (*n* = 3) of the AMD eyes showed evidence of a RPE tear. The associated PED subtypes were serous PED (*n* = 2) and haemorrhagic PED (*n* = 1).
- Drusen: Among the AMD eyes, 56 eyes (70.9%) showed presence of soft drusen. subretinal drusenoid deposits were detectable in 15 eyes (19.0%). Cuticular drusen were identified in 42 eyes (53.2%). Hard drusen were observed in 4 eyes (5.1%).

## Discussion

PEDs occur in association with various chorioretinal diseases and include drusenoid, serous, vascularized, mixed, and haemorrhagic subtypes based on previous classification systems [14, 49, 50]. The broad spectrum of possible aetiologies as well as different pathophysiological concepts emphasise PED being a non-specific clinical sign. PEDs may impair structural integrity of the photoreceptor-RPE-choriocapillaris complex with subsequent malfunctioning exchange of nutrients, oxygen, and bioactive molecules and, ultimately, functional impairment.

In clinical practice multimodal imaging assessment of PED is important for precise evaluation and optimal treatment approach. In this retrospective, cross-sectional, descriptive study, we focused on FAF imaging by confocal scanning laser ophthalmoscopy to further characterize PEDs with a horizontal diameter ≥350 µm. In general, alterations of the normal FAF distribution derive from absorbing factors, changes of the LF and MLF content and the viability of the RPE cell monolayer as well as from other fluorophores of the outer retina and the subneurosensory space [30, 33, 51–53]. The strongest FAF signal using 488 nm excitation wavelength is caused by the RPE. Using different excitation wavelengths, ex vivo autofluorescence from BM and sub-RPE deposits has been shown, whereas at 488-nm excitations, BM and sub-RPE deposits in normal eyes exhibited only minimal autofluorescence [54–56]. In the context of CSCR other possible fluorophores within the SRF are discussed in presence of debris and unphagocytosed photoreceptor outer segments [57–59].

The current study helps to catalogue possible changes of the FAF signal overlying PEDs. The findings reported herein underline the clinical variability and spectrum of morphological features of PEDs. The most frequent FAF changes within the area of PED were an irregular/granular FAF appearance followed by a homogeneously increased FAF signal. Only few PEDs were associated with decreased or normal FAF. Until now, these features are not readily explained, and interpretation remains partly speculative as proof by spectrophotometric analyses of all possible fluorophores within outer retinal layers, within the PED itself, and in presence of fluid (intraretinal, subretinal or sub-RPE) is lacking. In this study comparative SD-OCT imaging revealed pronounced RPE alterations in presence of PEDs contributing to changes of the FAF signal corresponding to the PED. SD-OCT scans indicated anterior RPE migration overlying the PED, i.e., anteriorly sloughed RPE and intraretinal hyperreflective foci, more frequently in association with irregular/granular FAF changes than in presence of homogeneously increased FAF. The FAF signal additionally was altered by presence of sub- or intraretinal fluid, haemorrhage, or RPE atrophy of different grades. A homogeneously increased FAF signal may possibly indicate an increased metabolic activity of the RPE monolayer, retinal thinning, and loss of macular pigments. Furthermore, partial loss of absorbing effects by photopigments in photoreceptors as well as possible fluorophores within the PED content need to be considered. Especially in cases with serous PEDs, fluorophores segregated by the RPE and possibly not viewable on SD-OCT scans may contribute to the altered FAF signal. In contrast to homogeneously increased FAF, an irregular/granular FAF signal is a mixed phenotypic appearance of increased, decreased, and normal FAF overlying the PED concomitant with the most extensive RPE alterations seen in SD-OCT. The described changes may result from secondary gradual RPE dysfunction, which, in chronic disease, shows RPE activation and anterior migration as a precursor of RPE and outer retinal atrophy [48, 60]. As seen in quantitative FAF as well as in histology, decreased autofluorescence may be indicative of fluorophore expulsion (LF/MLF) of the RPE and in presence of a long-standing lesion, leading to RPE cell dysfunction and finally RPE cell death [52, 61–63]. Another reason for slightly decreased FAF can be absorbing effects anterior to the RPE. Rarely, PEDs present with normal FAF in case of small PEDs with little or no affection of other retinal layers.

PEDs typically occur at the posterior pole with involvement of the foveal retina. Due to absorption properties of macular pigments, i.e., lutein and zeaxanthin, the foveal FAF signal is reduced in normal eyes [64–66]. In presence of subfoveal PED, this central decreased FAF signal was frequently changed leading to a displacement and compacting/condensation of the reduced central FAF signal. This may be due to retinal distension and dislocation of luteal pigment. However, a more detailed analysis of macular pigment changes requires further measurements of macular pigment optical density and macular pigment optical volume [67], which was not conducted in this study.

Associated features in presence of focally increased FAF overlying the PED included focal hyperpigmentations and subretinal accumulation of vitelliform material. Assuming that hyperreflective foci in OCT represent clusters of RPE cells, corresponding focally increased FAF seems to be likely [60]. Similarly, acquired vitelliform lesions come along with increased FAF, as they consist of RPE organelles like exploded LF/MLF granules, outer segment debris and RPE cell bodies [48, 60, 68]. Besides, an internal increased metabolic activity within the RPE can be assumed.

Hence, in presence of PEDs, overlying retinal layers frequently show structural alterations with impact on the FAF signal. Assumingly, these FAF changes do not necessarily correspond with the underlying PED subtype or a specific pathology. In OCT scans, drusenoid PEDs are mainly associated with a predominantly hyperreflective signal, whereas serous PEDs are more likely to appear predominantly optically empty. There is no preferred PED content in OCT associated with mixed PEDs, which may reflect the possible heterogeneous and variable composition of mixed PEDs. But there is no reliable conclusion regarding the PED content in OCT and the overlying FAF signal as it mainly derives from more anterior layers.

Separate from FAF changes overlying the PED, a FAF phenomenon was observed at the margin of many PEDs: a halo of decreased FAF, which was found in eyes with drusenoid, serous or mixed PEDs but did not occur in vascularized or haemorrhagic PEDs. A small amount of accompanying SRF at the PED’s margin in early stages may lead to absorption of the excitation light and the underlying RPE autofluorescence signal. Absorption effects by SRF are known from CSCR in the early phase [69, 70]. In contrast, in our study SRF was not required for the presence of the described halo. We, more likely, hypothesize an association with the steepness of the RPE elevation, which is more abrupt in eyes with serous or drusenoid PED compared to vascularized PEDs. The overlying retinal layers compensate the steep rise of the RPE resulting in a smooth elevated retinal surface with enlarged retinal thickness, and therefore increased absorption of the excitation light and the RPE’s FAF signal within the transition zone. Of course, additional SRF accumulation overlying the PED partially or completely interferes with FAF changes due to PED. Thereby, recent SRF can diminish the FAF signal, whereas in case of long-standing and chronic SRF other fluid fluorophores, like unphagocytosed photoreceptor outer segments, may contribute to an attenuated FAF signal [57]. Prior exudation may also interfere with FAF changes due to PED as increased FAF can be observed within an area of outer retinal atrophy and photoreceptor loss in terms of a “floodplain” [71].

Due to the horizontal and retrospective design various limitations of this study need to be considered. Examination was performed at one date, and we did not assess longitudinal variations over time regarding the FAF pattern. Since PEDs are expected to be dynamic during course of the disease and after therapy, temporal changes may occur during follow-up. These include changes of the PED morphology as well as associated secondary changes, which may affect and change the FAF pattern over time (e.g., RPE migration, RPE atrophy, accumulation of vitelliform material, new onset, or resorption of intra- and subretinal fluid). Additionally, the long-term prognostic significance of the described FAF changes needs further assessment in longitudinal studies. In general, extensive FAF changes are a clinically unpropitious sign with respect to retinal function [72, 73]. But at this juncture, the prognostic information still needs to be shown. In addition, the cross-sectional study design, and the large proportion of drusenoid PEDs may explain the small number of RPE tears, that is lower than in other studies [19, 74, 75]. Furthermore, lack of OCT angiography may result in limited ability to identify subretinal neovascularization, especially non-exudative quiescent macular neovascularization [16, 18, 76, 77]. Finally, this analysis was based on blue-light confocal scanning laser ophthalmoscopy FAF imaging. Use of non-confocal setups or other excitation/emission wavelengths for FAF imaging might reveal different results. Therefore, further prospective, and ideally longitudinal studies are needed for precise *and* simultaneous correlation of FAF changes with other imaging modalities, especially with SD-OCT, and OCT angiography.

Association of the FAF phenotype with the PED content as revealed in SD-OCT also holds some limitations. SD-OCT imaging might not be able to image deeper portions of the PED adequately. Hyporeflectivity may correspond with a fluid-filled portion of the PED but may be pretended by the limited ability to image deeper layers. Using enhanced depth imaging (EDI) OCT, Spaide found that PEDs may display an internal structure [20]. Therefore, from our point of view, in future clinical trials, combined and simultaneous FAF/EDI-OCT imaging would help to precisely correlate FAF and OCT findings.

In summary, FAF imaging of PEDs complements other imaging modalities. PEDs show a complex variety of alterations in FAF images which may reflect that PED is a non-specific sign for a spectrum of diseases and underlying causes. Changes of normal FAF within the area of PED rather derive from attenuation of overlying retinal layers (e.g., diminution or augmentation of absorbing factors, altered RPE cell LF/MLF content, anterior RPE migration, outer segment debris) than from the PED content itself.

### Summary Table

#### What was known before

- PEDs are a non-specific clinical sign of various chorioretinal diseases.
- PEDs can lead to FAF changes.
- Confocal scanning laser ophthalmoscopy is a convenient tool to record blue-light FAF.

#### What this study adds

- Most PEDs are associated with an irregular/granular or increased FAF signal.
- Morphologic changes within all retinal layers can contribute to complex FAF changes that do not follow a uniform pattern.
- FAF imaging gives additional information to better evaluate PED in the context of various chorioretinal diseases.

## Data Availability

All data generated or analysed during this study are included in this published article. Further inquiries can be directed to the corresponding author.
